# Human Respiratory Syncytial Virus NS 1 Targets TRIM25 to Suppress RIG-I Ubiquitination and Subsequent RIG-I-Mediated Antiviral Signaling

**DOI:** 10.3390/v10120716

**Published:** 2018-12-14

**Authors:** Junsu Ban, Na-Rae Lee, Noh-Jin Lee, Jong Kil Lee, Fu-Shi Quan, Kyung-Soo Inn

**Affiliations:** 1 Department of Fundamental Pharmaceutical Science, Graduate School, Kyung Hee University, 26 Kyungheedae-ro, Dongdaemun-gu, Seoul 130-701, Korea; mbjs3473@khu.ac.kr (J.B.); nrl9758@khu.ac.kr (N.-R.L.); minikyle0227@gmail.com (N.-J.L.); jklee3984@gmail.com (J.K.L.); 2 Department of Medical Zoology, School of Medicine, Kyung Hee University, 26 Kyungheedae-ro, Dongdaemun-gu, Seoul 130-701, Korea

**Keywords:** respiratory syncytial virus, nonstructural protein 1, RIG-I, TRIM25, interferon

## Abstract

Respiratory syncytial virus (RSV) causes severe acute lower respiratory tract disease. Retinoic acid-inducible gene-I (RIG-I) serves as an innate immune sensor and triggers antiviral responses upon recognizing viral infections including RSV. Since tripartite motif-containing protein 25 (TRIM25)-mediated K63-polyubiquitination is crucial for RIG-I activation, several viruses target initial RIG-I activation through ubiquitination. RSV NS1 and NS2 have been shown to interfere with RIG-I-mediated antiviral signaling. In this study, we explored the possibility that NS1 suppresses RIG-I-mediated antiviral signaling by targeting TRIM25. Ubiquitination of ectopically expressed RIG-I-2Cards domain was decreased by RSV infection, indicating that RSV possesses ability to inhibit TRIM25-mediated RIG-I ubiquitination. Similarly, ectopic expression of NS1 sufficiently suppressed TRIM25-mediated RIG-I ubiquitination. Furthermore, interaction between NS1 and TRIM25 was detected by a co-immunoprecipitation assay. Further biochemical assays showed that the SPRY domain of TRIM25, which is responsible for interaction with RIG-I, interacted sufficiently with NS1. Suppression of RIG-I ubiquitination by NS1 resulted in decreased interaction between RIG-I and its downstream molecule, MAVS. The suppressive effect of NS1 on RIG-I signaling could be abrogated by overexpression of TRIM25. Collectively, this study suggests that RSV NS1 interacts with TRIM25 and interferes with RIG-I ubiquitination to suppress type-I interferon signaling.

## 1. Introduction

Respiratory syncytial virus (RSV) belongs to the family *Pneumoviridae* and contains a negative-sense single-stranded RNA genome. RSV infection is a leading cause of severe acute lower respiratory tract disease and related hospitalization in children and the elderly [1,2]. Despite the global burden from RSV infection, there are no available RSV-specific vaccines or effective therapeutic agents at present.

The cellular innate immune system utilizes various sensors including Retinoic acid inducible gene-I (RIG-I) and Toll-like receptors (TLRs) to detect viral infection and activate antiviral immune signaling pathways [3,4]. Among these, RIG-I and Toll-like receptor 3 (TLR3) have been implicated in early antiviral immune responses against RSV infection in airway epithelial cells [5]. Deficiency of mitochondrial antiviral signaling protein (MAVS) and myeloid differentiation primary response (MyD88), which are crucial downstream molecules of RIG-I signaling and TLR signaling, respectively, resulted in an increased viral load in mice, indicating the role of RIG-I and TLR signaling pathways against RSV infection [6]. RIG-I, a cytoplasmic RNA-sensing molecule, has been well-known to trigger antiviral signaling, including the production of type-I interferon [7]. RIG-I comprises 2 N-terminal caspase recruitment domains (CARDs), a helicase domain and a C-terminal regulatory domain (CTD). Upon recognition of viral RNAs by the helicase and CTD domain of RIG-I, the two N-terminal CARDs are exposed and interact with TRIM25, a E3-ubiquitin ligase. TRIM25 delivers the K63-linked polyubiquitin chain to the RIG-I CARDs to induce RIG-I oligomerization and subsequent interaction with MAVS [8]. Since TRIM25 plays a crucial role in RIG-I signaling pathways, several viral proteins target TRIM25 to evade RIG-I-mediated antiviral responses [9]. For example, NS1 of influenza A virus interacts with TRIM25 to inhibit its oligomerization and subsequent RIG-I ubiquitination [10].

Like other viruses, RSV has evolved several strategies to antagonize antiviral immune responses [11]. The role of Nonstructural Proteins 1 and 2 (NS1 and NS2) in suppressing innate immune signaling has been particularly highlighted [12,13,14]. The RSV NS2 protein has been shown to interact with RIG-I CARDs to suppress RIG-I signaling [15]. NS1 protein is also known to co-localize with MAVS, suggesting its role in suppressing RIG-I-mediated antiviral responses [16]. Interestingly, the NS1 and NS2 of RSV are capable of inducing the degradation of key molecules in RIG-I signaling and type-I interferon signaling including RIG-I, IRF3, IRF7, TBK1 and STAT2, to suppress antiviral responses by forming a large degradative complex [17]. In the previous study, ectopic expression of NS1 or NS2 sufficiently inhibited RIG-I-mediated interferon production, indicating that NS1 is also capable of suppressing RIG-I signaling by itself like NS2 [18]. However, the underlying mechanism and molecular target of NS1-mediated RIG-I signaling inhibition remain elusive yet. In the current study, we explored the possibility that NS1 proteins of RSV interfere with TRIM25-mediated RIG-I ubiquitination to dissect the molecular mechanism underlying the direct inhibition of RIG-I signaling by NS1/2 of RSV.

## 2. Materials and Methods

### 2.1. Cells, Virus and Plasmids

HEK293T, HEp-2 (Human epithelial type 2) and A549 (human alveolar basal epithelial cells) cells were maintained in Dulbecco’s High Glucose Modified Eagle’s Medium (DMEM; Hyclone, Logan, UT, USA) containing 10% fetal bovine serum (FBS; Hyclone) and 1% penicillin/streptomycin (Hyclone).

RSV A2 strain stocks were prepared as described previously [19]. Briefly, HEp2-cells were infected with RSV and incubated for 5 days. After lysis viral particles were harvested by ultracentrifugation. The viral titer was determined by plaque assay as described previously [19].

pEBG-GST-RIG-IN (N-terminal 2 Cards domain of RIG-I), pEF-IRES (pIRES)-V5-TRIM25 and MAVS-CARD-proline rich domain (PRD)-FLAG were described in a previous study [20]. pIRES-V5 -RING, pIRES-V5-B-box/CCD, pIRES-V5-SPRY and pIRES-V5-SPRY deletion mutants were used previously [8]. We obtained the RSV NS1 and NS2 sequences from GenBank (GenBank KT992094.1) to synthesize codon-optimized RSV NS1 and NS2 for sufficient expression in mammalian cells. RSV NS1 and NS2 were cloned into the pCDNA 3.1 vector at the restriction enzyme site between BamHI and XbaI with a C-terminus FLAG tag and HA tag, respectively. Additionally, RSV NS1 was inserted into the pEF-HisA-V5 plasmid between the BamHI and NotI restriction sites.

### 2.2. Transfection and Reagents

Conventional calcium phosphate transfection and polyethylenimine (PEI) transfection methods were used for transfection. The total plasmid content for each sample condition was adjusted using the empty vector. Low molecular weight polyI:C (Invivogen, San Diego, CA, USA); average size from 0.2kb to 1kb) was transfected with PEI solution to mimic intracellular RNAs.

### 2.3. Co-Immunoprecipitation (Co-IP) and GST-Pulldown Assay

Cells were collected and lysed in Triton X-100 cell lysis buffer (50 mM Tris-HCl, 150 mM NaCl, 5mM EDTA, 0.1% Triton X-100) containing protease and phosphatase inhibitor cocktail (Thermo Scientific, Waltham, MA, USA). Lysates were incubated overnight at 4 °C with the corresponding antibody. After the binding reaction, protein A/G resin (Sigma, St. Louis, MO, USA) were added to samples and further incubated for 2 h at room temperature. The resin was then washed four to five times with cell lysis buffer. Anti-FLAG antibody conjugated resin (Sigma) and anti-V5 antibody conjugated resin (Sigma) were also used for Co-IP in a similar manner. Precipitated proteins were eluted with SDS sample buffer (250 mM Tris-HCl, 10% SDS, 30% glycerol, 25% mercaptoethanol, 0.05% bromophenol blue) and subjected to SDS-PAGE and immunoblotting.

To precipitate GST-tagged fusion proteins, the cell lysates were incubated with glutathione-conjugated beads (Sigma) at RT for 1–2 h. After incubation, the beads were washed four to five times with cell lysis buffer, followed by SDS-PAGE and immunoblotting.

### 2.4. Immunoblotting

After polyacrylamide gel electrophoresis, target proteins on the gel were transferred to a PVDF membrane (Millipore, St. Charles, MO, USA). The membrane was incubated overnight at 4 °C with the indicated antibody in 5% bovine serum albumin (BSA, RMBio, Missoula, MT, USA) solution for binding. After extensive washing with PBST, the membrane was then incubated with the horseradish peroxidase (HRP) conjugated secondary antibody at RT for 2 h. Luminata Forte (Millipore) was used as the HRP substrate. Anti-V5 rabbit antibody (Cell signaling, Denvers, MA, USA, #13202), anti-V5 mouse antibody (E-bioscience, San Diego, CA, USA, #14-6796-80), anti-HA mouse antibody (SantaCruz, Santa Cruz, CA, USA, #sc-7392), anti-FLAG mouse antibody (Sigma, #F7425), anti-ubiquitin (P4D1) mouse antibody (Cell signaling, #3936), anti-GST antibody (Abcam, Cambridge, UK, #19256) and HRP conjugated antibodies (Cell signaling, #7074, #7076) were used.

### 2.5. Luciferase Assay

HEK293T cells in 24-well plates were transfected with Interferon-β firefly luciferase (0.25 μg/well) and thymidine kinase (TK) renilla reporter plasmids (0.13 μg/well) were transfected along with other constructs as indicated [21]. After 24 h, transfected HEK293T cells were lysed in passive lysis buffer (Promega, Madison, WI, USA) at RT for 15 min. Cell lysates were then analyzed by a dual-luciferase assay according to the instruction (Promega). All assays were performed in triplicate and repeated at least three times. 

### 2.6. Reverse-Transcription Quantitative Polymerase Chain Reaction (RT-qPCR)

Total RNAs were extracted using TRIzol (Thermo Scientific, #15596018) according to the instruction. cDNAs were generated from RNAs (1 μg) using Superscript III reverse transcriptase (Thermo Scientific, #18080093) and oligo_20_(dT) primers. Real-time PCR was conducted using synthesized cDNA (2 μL). The mRNA levels of interferon-β and interferon-stimulated gene 15 (ISG15) were determined using the following primers and normalized to those of β-actin: interferon-β-forward; 5′-AAGAGTTACACTGCCTTTGCCATC-3′, interferon-β-reverse; 5′-CACTGTCTGCTGGTGGAGTTCATC-3′, ISG15-forward; 5′-CCTCTGAGCATCCTGGT-3′, ISG15-reverse; 5′-AGGCCGTACTCCCCCAG-3′, β-actin-forward; 5′-TGGAATCCTGTGGCATCCATGAAAC-3′, β-actin-reverse; 5′-TAAAACGCAGCTCAGTAACAGTCCG-3′.

### 2.7. Confocal Microscopy

HEK293T cells were seeded onto fibronectin coated coverslips in a 24-well plate. After 24 h, cells were transfected with FLAG-tagged RSV NS1 and V5-tagged TRIM25 and incubated for 24 h. Then, cells were fixed with 4% paraformaldehyde followed by blocking and permeabilization in permeabilization buffer (0.5% BSA, 0.2% triton X-100 in PBS) at RT in 5 min. The cells were incubated with anti-FLAG mouse and anti-V5 rabbit antibodies in 0.5% BSA solution at 4 °C overnight. Next day, the cells were extensively washed and incubated with FITC- and PE-labeled secondary antibodies at RT for 2 h. The co-localization between RSV NS1-FLAG and TRIM25-V5 was analyzed by Nanoscope K1-Fluo confocal microscope. 

### 2.8. Statistical Analysis

Data were presented as the mean ± SEM. Statistical comparisons between the control and treated groups were performed using the Student’s *t*-test. A value of *p* ≤ 0.05 was considered to be significant.

## 3. Results

### 3.1. RSV NS1 Inhibits RIG-I-Mediated Interferon Signaling

**Figure 1:**
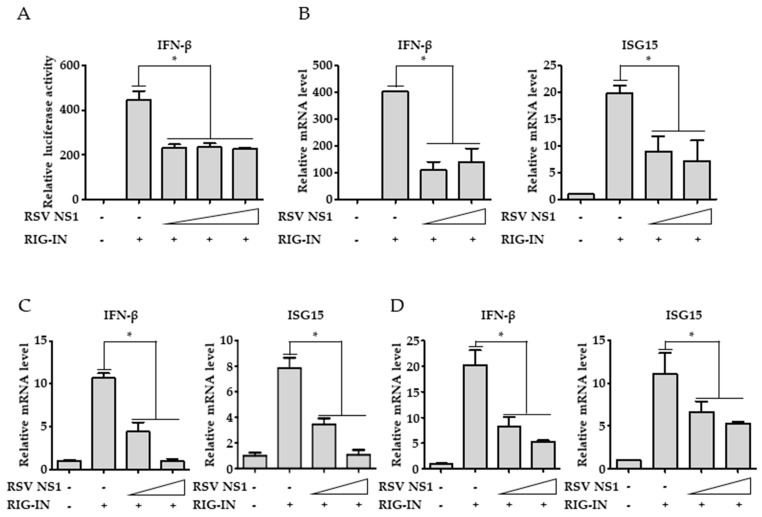
Suppression of RIG-I dependent interferon signaling by RSV NS1. (**A**) HEK293T cells were transfected with RIG-I-2Cards (RIG-IN; 10 ng/well) together with increasing amount of RSV NS1 (63, 125, 250 ng/well) as indicated together with Interferon (IFN)-β promoter firefly luciferase and Thymidine kinase (TK) renilla luciferase reporter plasmids. Promoter activities were determined by dual-luciferase assays. Data were presented as the mean ± SEM. * *p* < 0.05. (**B**–**D**): HEK293T (**B**) A549 (**C**) and HEp-2 (**D**) cells were transfected with RIG-IN together with increasing amounts of NS1 (63 and 250 ng/well). Total RNAs were prepared 24 h after transfection and subjected to RT-qPCR to determine mRNA levels of IFN-β and ISG15. Data were presented as the mean ± SEM. * *p* < 0.05. All experiments were repeated at least three times. The results show the most representative data from a single experiment conducted in triplicate.

To confirm the suppression of RIG-I-mediated signaling by NS1, RSV NS1 was transfected along with constitutively active RIG-I-2CARDs (RIG-IN). Ectopically expressed NS1 inhibited interferon-β promoter activity that was induced by RIG-IN as determined by the luciferase assays in HEK293T cells, confirming that NS1 itself is capable of inhibiting RIG-I-mediated antiviral signaling (Figure 1A). Consistently, RIG-IN-mediated induction of interferon-β and ISG15 mRNA synthesis was significantly hampered by ectopic expression of NS1 (Figure 1B). Similar results were obtained from experimental settings using A549 and HEp-2 cells, suggesting that NS1 is able to suppress RIG-I-mediated type-I interferon production in various cells including airway epithelial cells (Figure 1C,D).

### 3.2. RSV NS1 Suppresses TRIM25-Mediated RIG-I Ubiquitination

**Figure 2:**
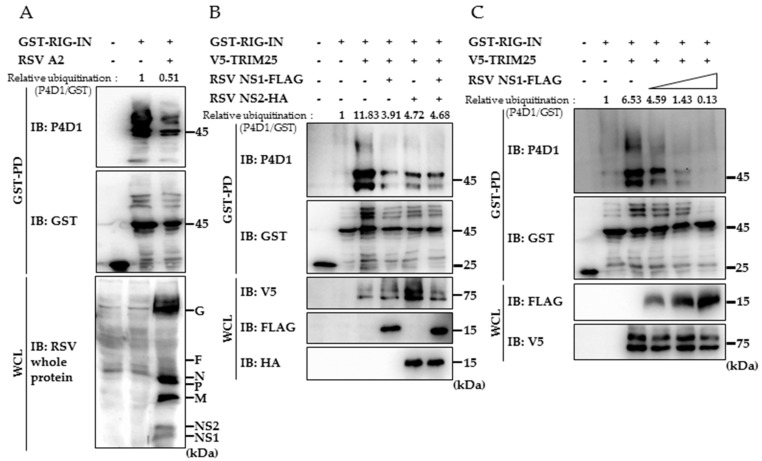
Inhibition of RIG-I ubiquitination by RSV NS1. (**A**) HEK293T cells were transfected with vector (pEBG-GST; 7 μg/dish) or GST-RIG-I-2Cards (GST-RIG-IN; 10 μg/dish) and incubated for 24 h. Cells were infected with RSV (m.o.i. = 4) as indicated and further incubated for 24 h. Cell lysates were subjected to GST-pulldown and immunoblotting using indicated antibodies to analyze the ubiquitination of GST-RIG-IN. Ubiquitinated forms were detected using anti-ubiquitin antibody (P4D1). (**B**) RSV NS1 and NS2 expression plasmids were transfected to HEK293T cells together with RIG-IN and TRIM25 as indicated. Cell lysates were subjected to GST-pulldown assays. Ubiquitination of RIG-IN was analyzed by immunoblotting of GST pulldown samples and whole cell lysates (WCL). (**C**) RIG-IN and TRIM25 were transfected with increasing amounts of RSV NS1. RIG-IN ubiquitination was analyzed as in (**B**). Ubiquitination of RIG-I was quantitatively analyzed by densitometry and normalized to pulled-down GST-RIG-IN (unubiquitinated form) of each sample. All experiments were conducted at least three times with similar results.

Because TRIM25-mediated RIG-I CARD ubiquitination is an essential step for the successful induction of RIG-I-mediated antiviral responses, we explored the possibility that RSV suppresses RIG-I activation by inhibiting the TRIM25-mediated RIG-ubiquitination. First, we have tested whether RIG-I ubiquitination is suppressed by RSV infection. As seen in Figure 2A, ubiquitination of ectopically expressed GST-RIG-IN is decreased by the presence of RSV (multiplicity of infection = 4), suggesting that RSV is capable of suppressing RIG-I ubiquitination to modulate RIG-I signaling. Next, we have tested whether RIG-I ubiquitination is affected by the presence of NS1. As shown in Figure 2B, both NS1 and NS2 suppressed the TRIM25-mediated ubiquitination of RIG-IN. Increasing amounts of NS1 resulted in an augmented effect on reducing the ubiquitinated form of RIG-IN (Figure 2C). The results clearly showed that NS1 is capable of interfering with TRIM25-mediated RIG-I ubiquitination to suppress RIG-I-mediated interferon production.

### 3.3. RSV NS1 Protein Interacts with TRIM25

**Figure 3:**
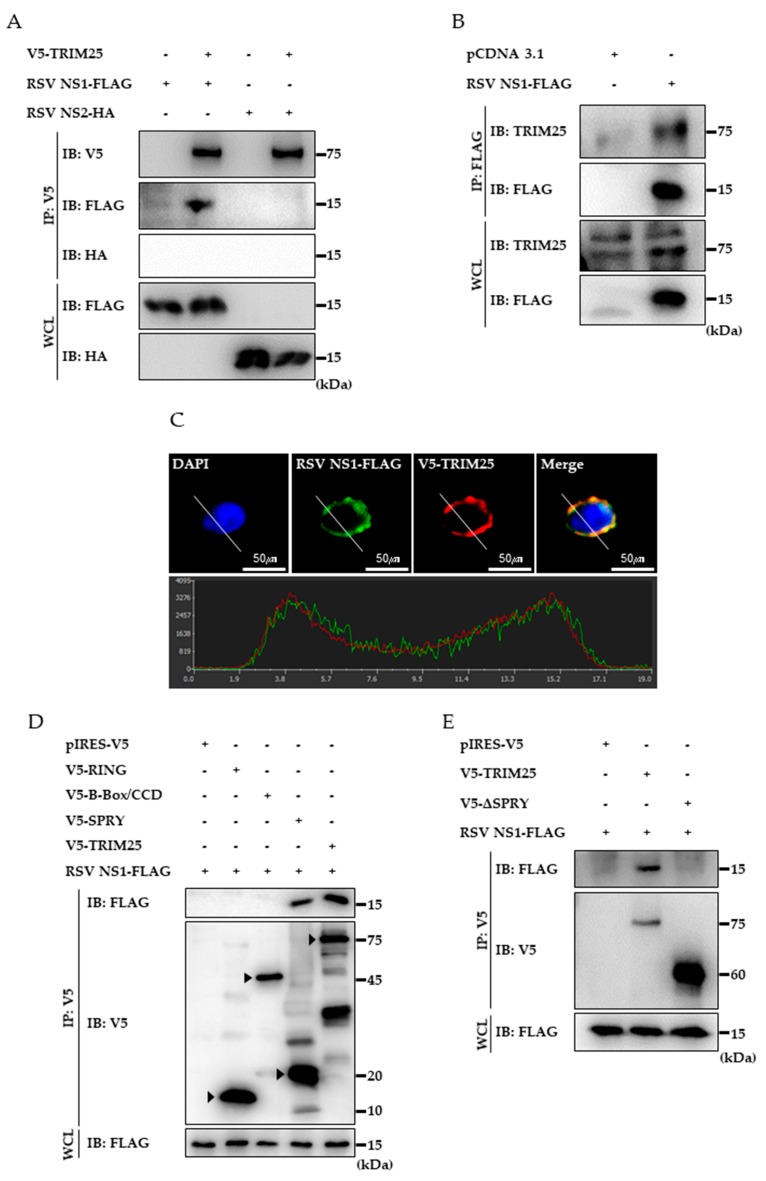
Interaction between RSV NS1 and TRIM25. (**A**) To test whether RSV NS proteins interact with TRIM25, V5-TRIM25 was expressed with RSV NS1-FLAG or NS2-HA in HEK293T. Whole cell lysates (WCL) were subjected to co-immunoprecipitation (co-IP) and immunoblotting using indicated antibodies. (**B**) FLAG-tagged RSV NS1 was overexpressed in HEK293T. Cell lysates were immunoprecipitated with anti-FLAG antibody and analyzed by immunoblotting. (**C**) HEK293T cells were transfected with FLAG-tagged NS1 and V5-tagged TRIM25 expression plasmids. Localization of NS1 and TRIM25 were visualized by primary antibodies and FITC- and PE-labeled secondary antibodies as described in Material and Methods. The lower panel shows the intensity of NS1 (green) and TRIM25 (red) along the white line of the upper panel images. (**D**) TRIM25 truncated mutants including V5-tagged RING, B-box/CCD and SPRY domain constructs were expressed in HEK293T cells together with RSV NS1. Co-IP was performed followed by immunoblotting. The arrowheads indicate the bands with correct size of each construct. (**E**) Wild type TRIM25 or SPRY deletion (ΔSPRY) mutant plasmid was transfected with RSV NS1-FLAG followed into HEK293T cells. Interaction was analyzed by co-IP and immunoblotting as indicated. All experiments were conducted at least three times with similar results.

Interaction between RIG-IN and NS1 was examined using a GST-pulldown assay to test whether NS1 interacts with RIG-IN similar to NS2. However, we could not detect any interaction (Data not shown). Therefore, interaction between TRIM25 and NS1 was investigated to determine whether NS1 targets TRIM25 to suppress RIG-I ubiquitination. Obvious interaction between ectopically expressed TRIM25 and NS1 was detected by the co-immunoprecipitation assay, whereas interaction between NS2 and TRIM25 was not detected (Figure 3A). Furthermore, endogenous TRIM25 was also co-precipitated with NS1, supporting that RSV NS1 targets TRIM25 (Figure 3B). Co-localization of NS1 and TRIM25 in the cytoplasm was detected by confocal microscopic observation (Figure 3C). To further dissect the interaction between NS1 and TRIM25, we determined the domain of TRIM25 that is responsible for this interaction using truncated TRIM25 domains. FLAG-tagged NS1 was expressed along with the RING domain, B-Box/CCD domain, or SPRY domain of TRIM25 and co-immunoprecipitation assays were performed. As depicted in Figure 3D, the SPRY domain sufficiently interacts with NS1, whereas the RING or B-Box/CCD domains do not show any interaction. In addition, the SPRY deleted TRIM25 mutant failed to interact with NS1, indicating that the SPRY domain is required for interaction with NS1 (Figure 3E).

### 3.4. RSV NS1 Protein Hinders RIG-I 2CARD Interaction with MAVS CARD

**Figure 4:**
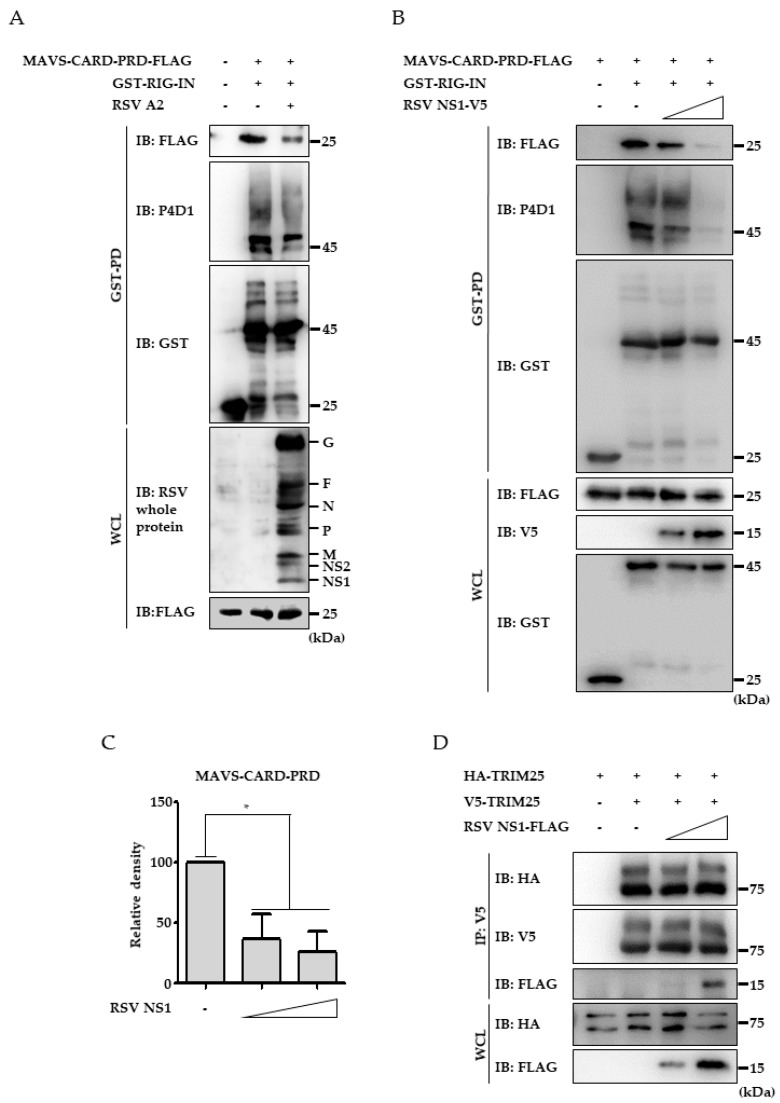
Inhibition of RIG-IN interaction with MAVS-CARD-PRD by RSV NS1. (**A**) GST-RIG-IN or GST vector was transfected to HEK293T cells together with MAVS-CARD-PRD-FLAG plasmid as indicated. After 24h, cells were infected with RSV (moi=4) as indicated and further incubated for 24 h. Cell lysates were subjected to GST-pulldown and immunoblotting using indicated antibodies to analyze the ubiquitination of GST-RIG-IN. (**B**) MAVS-CARD-PRD-FLAG, RSV NS1-V5 and GST-RIG-IN or GST vector plasmids were transfected into HEK293T cells as indicated. The lysates (WCL) were analyzed by co-IP and immunoblotting. Ubiquitinated forms were detected using anti-ubiquitin antibody (P4D1). (**C**) Densitometric analysis of the precipitated MAVS-CARD-PRD-FLAG. The levels of co-precipitated MAVS-CARD-PRD-FLAG were analyzed by densitometry using Vision-Capt software and normalized to the levels of pulled-down GST-RIG-IN. (**D**) HA- and V5-tagged TRIM25 (10 μg/Φ10 cm dish) with or without FLAG-tagged NS1 (0, 4 or 8 μg/Φ10 cm dish) were transfected into HEK293T cells as indicated. Interaction between HA-TRIM25 and V5-TRIM25 was analyzed by co-IP and immunoblotting using indicated antibodies. All experiments were conducted at least three times with similar results.

Because RIG-I ubiquitination is prerequisite for interaction with MAVS, decreased RIG-I CARD ubiquitination by RSV NS1 should result in disruption of RIG-I interaction with MAVS. Thus, the interaction between RIG-IN and MAVS upon virus infection was analyzed. As shown in Figure 4A, clear interaction between FLAG-tagged MAVS-CARD-proline rich domain (PRD) and GST-tagged RIG-IN was detected by co-IP. However, RSV infection resulted in decrease of both RIG-IN ubiquitination and the interaction between RIG-IN and MAVS-CARD, supporting that RSV is capable of suppressing RIG-I ubiquitination and the subsequent RIG-I interaction with MAVS (Figure 4A). To further confirm the effect of NS1 on RIG-I activation, the effect of NS1 ectopic expression on RIG-IN interaction with MAVS was examined. FLAG-tagged MAVS-CARD-PRD and GST-tagged RIG-IN was co-expressed with increasing amounts of V5-tagged NS1 followed by a co-immunoprecipitation assay using an anti-FLAG antibody. As seen in Figure 4B,C, the clear interaction between RIG-IN and MAVS-CARD-PRD was decreased by NS1 expression in a dose-dependent manner. These results suggest that RSV NS1 expression diminishes the interaction between RIG-I and MAVS by interfering with TRIM25-mediated RIG-I ubiquitination. Since TRIM25 oligomerization is crucial for its E3-ligase activity, it has been tested whether NS1 suppresses TRIM25 E3-ligase activity by interfering the oligomerization. As seen in Figure 4D, expression of NS1 did not affect the interaction between HA-tagged TRIM25 and V5-tagged TRIM25 while interaction between V5-tagged TRIM25 and NS1 was detected. It indicates that decreased RIG-I ubiquitination by NS1 is not due to the inhibition of TRIM25 oligomerization.

### 3.5. Ectopic Expression of TRIM25 Reverses the Effect of NS1 on RIG-I-Mediated Interferon Signaling

**Figure 5:**
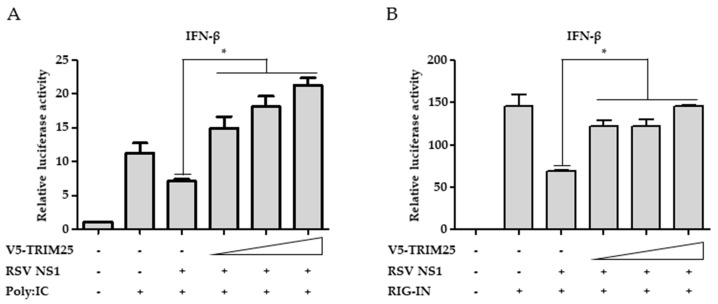
Ablation of interferon-β promoter suppressive effect of NS1 by ectopic expression of TRIM25. (**A**,**B**) RSV NS1 was expressed with increasing amount of TRIM25 in HEK293T cells. To induce interferon-β promotor activity, low molecular weight polyI:C (**A**) or RIG-IN (**B**) were transfected at the same time as indicated. Interferon (IFN)-β promoter firefly luciferase and Thymidine kinase (TK) renilla luciferase reporter plasmids were co-transfected. Interferon-promoter activities were analyzed by luciferase assays. Data were presented as the mean ± SEM. * *p* < 0.05. The experiments were repeated at least three times. The results show the most representative data from a single experiment conducted in triplicate.

To confirm that interaction between NS1 and TRIM25 contributes to the interferon-suppressive effect of NS1, the effect of TRIM25 overexpression on the suppression of RIG-I signaling by NS1 was investigated using interferon-β luciferase promoter assays. As shown in Figure 5A, activation of interferon-β promoter activity by polyI:C transfection was suppressed by NS1 expression. The effect of NS1 was abrogated by the overexpression of TRIM25 (Figure 5A). Similarly, suppression of RIG-IN-induced interferon-β promoter activity by NS1 was also reversed by the ectopic expression of TRIM25 (Figure 5B). These results indicate that NS1 interaction with TRIM25 contributes to its RIG-I suppressive activity.

## 4. Discussion

Many viruses possess defensive mechanisms against the host immune system for their effective replication. The NS1 and NS2 proteins of RSV have been shown to effectively inhibit the host immune system. NS proteins target diverse proteins related to type-I interferon induction and signal transduction. For example, RSV NS1 upregulates SOCS1 and SOCS3 and triggers STAT2 degradation [12]. RSV NS1 also inhibits the interferon alpha response by targeting the interferon alpha receptor [13]. NS2 also degrades STAT2 to downregulate interferon-mediated JAK-STAT signaling responses [14]. In addition, NS1 and NS2 exert suppressive activities on the RIG-I-mediated antiviral signaling pathway, which is a crucial response against RSV infection. Previous studies suggest that NS1 and NS2 inhibit RIG-I-mediated signaling by inducing degradation of key molecules such as IRF3/7 [21,22].

In this study, we explored the possibility that these proteins interfere with RIG-I signaling by directly inhibiting RIG-I activation. Indeed, we could demonstrate that NS1 and NS2 suppressed TRIM25-mediated ubiquitination of RIG-I, which is a crucial step for RIG-I activation. Previously, NS2 has been shown to interact with RIG-I, whereas the interaction between NS1 and RIG-I could not be detected [15]. Thus, suppression of RIG-I ubiquitination by NS2 may be due to its interaction with RIG-I. We also could not detect interaction between NS1 and RIG-I, indicating that NS1 may utilize a separate molecular mechanism to suppress RIG-I ubiquitination. In addition, a previous study showed that NS1 co-localizes with MAVS and inhibits RIG-I interaction with MAVS [16]. Considering that RIG-I ubiquitination is required for its oligomerization and interaction with MAVS, it is conceivable that NS1 suppresses RIG-I interaction with MAVS by interfering with RIG-I ubiquitination, as shown in the current study.

Thus, it was tempting to test the hypothesis that NS1 targets TRIM25, an E3-ubiquitin ligase responsible for RIG-I ubiquitination. Moreover, several viruses utilize their proteins to interfere with TRIM25 activation and subsequent RIG-I activation. For instance, the Influenza NS1 binds to TRIM25 and inhibits TRIM25 multimerization [10]. The SARS coronavirus nucleocapsid protein binds to TRIM25 SPRY and disrupts RIG-I ubiquitination [23]. In this study, the interaction between NS1 and TRIM25 was demonstrated. Since several studies have shown that TRIM25 migrates to the mitochondria upon viral infection to interact with MAVS [24], the interaction between MAVS and NS1 might be a result of the interaction between NS1 and TRIM25. As expected, NS1 inhibited the interaction between RIG-I CARD and MAVS. These results clearly indicate that NS1 is capable of suppressing RIG-I activation by targeting TRIM25 and inhibiting RIG-I ubiquitination through TRIM25. We have shown that NS1 could suppress the interferon-β promoter activity induced by RIG-IN and MAVS, indicating that NS1 is also capable of suppressing downstream signaling. This can be explained by previous studies showing that NS1 can trigger the degradation of critical downstream molecules such as IRF3. Nonetheless, we have shown that NS1 can suppress TRIM25-mediated ubiquitination of RIG-IN without affecting its amount and that its effect on RIG-IN-mediated interferon-β promoter activity can be reversed by an excessive amount of TRIM25. These results indicate that inhibition of TRIM25-mediated RIG-I ubiquitination by NS1 contributes to the suppression of RIG-I signaling, at least in part.

Interestingly, the biochemical domain mapping study revealed that the SPRY domain of TRIM25 is responsible for interaction with NS1. Given that the SPRY domain is responsible for interaction with RIG-I, a possible molecular mechanism is that NS1 binds to TRIM25 and sequesters it to prevent its interaction with RIG-I. However, we could not observe a significant reduction in interaction between RIG-I and TRIM25 by NS1 (Data not shown). Further studies are needed to identify the detailed molecular mechanism of RSV NS1-mediated TRIM25 dysfunction.

RSV NS2 is known to bind with RIG-I CARD and disrupts its activation [15] and we showed that RSV NS1 interacts with TRIM25 to decrease CARD ubiquitination. In a previous study, RSV NS1 and NS2 have been shown to exist as homo- or heterodimers in mammalian cells. The RSV NS1-NS2 dimer was detected in mitochondria, suggesting that NS1 and NS2 can cooperate to suppress RIG-I signaling [18]. It is also possible that the dimer of RSV NS1 and NS2 interacts with TRIM25 and RIG-I CARD through each NS protein interacting with TRIM25 or RIG-I CARD. Although we could not observe a significant synergistic effect of NS1 and NS2 co-expression in terms of suppression of RIG-I ubiquitination (Figure 1C), the NS1 and NS2 interaction may have additional functions in RIG-I signaling. For instance, it is possible that the NS1/NS2 complex induces RIG-I degradation followed by suppression of RIG-I K63-ubiquitination for complete inhibition of the RIG-I signaling pathway.

Collectively, this study demonstrates that RSV NS1 interacts with TRIM25 and suppresses TRIM25-mediated RIG-I activation to evade RIG-I-mediated antiviral responses. Further studies including structural analysis of protein interaction and analysis of the interaction modes between RIG-I, TRIM25, NS1 and NS2 need to be conducted.
